# Characterizing the Role of *Moringa oleifera* Lam (MO) Leaves and Root Extracts on *Dictyostelium discoideum* Cell Behavior

**DOI:** 10.3390/biology14030284

**Published:** 2025-03-11

**Authors:** Sarah Abdulaziz Alamer, Fadia El Sherif

**Affiliations:** Department of Biological Sciences, College of Science, King Faisal University, Al-Ahsa 31982, Saudi Arabia; felsherif@kfu.edu.sa

**Keywords:** *Moringa oleifera* Lam, flavonoids, apigenin, cell growth, chemotaxis, *Dictyostelium discoideum*

## Abstract

*Moringa oleifera* Lam (MO) is a member of the Moringaceae family and has been widely used as a traditional form of treatment for various diseases due to its high nutrient content. The plant is rich in vitamins, minerals, organic acids, phenolic compounds, polyphenols, alkaloids, and flavonoids. However, the concentrations of these components in each part of the plant differ, leading to specific beneficial uses. In this study, we aimed to analyze the contents of *Moringa oleifera* leaf (ML) and *Moringa oleifera* root (MR) extracts and characterize the effects of these extracts on cell behavior. HPLC analysis data showed a higher level of flavonoids and apigenin in the ML extract compared to the MR extract. Furthermore, CG/MS analysis revealed 54 components in the ML extract, with only 3 (ethyl palmitate, ethyl linolenate, and palmitic acid, 2-(octadecyloxy)ethyl ester) of them being at high levels. In this study, *Dictyostelium discoideum* was used as a cellular model and *D. discoideum*’s cell growth, chemotaxis, and development life cycle were investigated. The data presented herein demonstrate a significant decrease in cell growth and that the completion of the development life cycle was delayed in the ML extract-treated sample. This effect was not found in the untreated cells and MR extract-treated samples. In addition, the ability of cells to stream during chemotaxis was not inhibited following treatments. These findings suggested that ML extract has an impact on cell proliferation and cell directed migration processes, where the high level of flavonoids and apigenin in this extract can be a strong factor that led to these results.

## Simple Summary

Age-related diseases, including cancer and neurological disorder, have been major targets for medical and pharmacological research for decades. Cell proliferation and cell chemotaxis are two important aspects in the development of such disorders. Among the natural products that were subjected to investigation for their therapeutic effect, Moringa oleifera Lam (MO), which is known as miracle tree, showed a positive impact on different diseases. This is due to the rich bioactive compounds the tree possesses. Our results have provided evidence of the different functions for different parts of the *M. oleifera* that grow in Al-Ahsa province, which is located in the Eastern region of Saudi Arabia.

## 1. Introduction

The Moringa genus is a member of the Moringaceae family, which consists of 13 different species, including *Moringa oleifera*, which has been widely used for novel applications in the fields of pharmacology and biomedicine [1]. The results of a previous study indicated that the plant shows different nutritional compositions in different countries [2]. Each part of this plant is rich in minerals, fibers, proteins, sugars, free proline, free amino acids, vitamins, and phytohormones, in addition to phenolic compounds that have potential health benefits, including anticancer and antioxidant activity [3,4].

The impact of Moringa extracts on human disease is a key area of interest in the scientific research field. The positive impact of this plant on cancer as an antiproliferative and antiangiogenic agent has been reported in previous studies. The application of extracts of Moringa leaf exhibited a significant effect on cell growth in both a mouse melanoma tumor model and A549 lung cells [5,6]. ROS accumulation in cholangiocarcinoma cells was observed after cells were treated with Moringa leaves and seed extracts, which led to cell apoptosis. Together, these results provide evidence of the plant’s anticancer activity [7]. However, the application of extracts from *M. oleifera* leaf collected from Thailand caused a reduction in ROS accumulation in HEK-293 cells [8]. These findings emphasize the need for further investigation into the compounds responsible for the observed biological effects.

Scientific discoveries over the last decade indicate the importance of this plant. Saudi Arabia is one of the indigenous origins of *M. oleifera*. Although 205 research papers on this subject were published in the KSA during the period of 2000–2022, there is no clear analysis of the active components of root and leaf extracts obtained from the *M. oleifera* plant, which grows in Saudi Arabia [9]. Considering data from previous studies in the *M. oleifera* field, researchers have yet to use *M. oleifera* that grows in the Al-Ahsa province. It is, therefore, imperative to explore the biological impact of *M. oleifera* extracts as therapeutic agents in greater depth. 

Over the past few decades, numerous studies have been conducted on the directed cell migration of amoeboid cells. Such studies have primarily involved the use of the lower eukaryotic amoeba *D. discoideum* as a model organism for studying cell behavior and chemotaxis [10]. This cellular model is characterized by a special life cycle consisting of two states, vegetative and development. In the vegetative phase, cells feed on bacteria and divide by mitosis. However, starvation initiates a developmental cycle based on the expression of different proteins, cells secreting cAMP, and the initiation of waves of cell aggregation that proceed to different stages until a multicellular fruiting body structure is formed [11,12]. This model has provided significant insights into cell growth, chemotactic behavior, development, and the common signals that are involved in these actions. These signals are mostly conserved in many cell types of higher eukaryotes, including amoeba-like neutrophils [13]. Different approaches can be applied with the use of this simple eukaryotic model. In addition, *D. discoideum* was employed as a pharmacological model to study the mechanisms of medicinal drugs and natural products [14].

Although the potential health benefits of moringa have already been determined, its role in *D. discoideum* cell growth and amoeboid migration remains undefined [15]. This remarkable model exhibits a fundamental phenomenon during the starvation phase whereby cells are able to be chemotactic toward cAMP. Chemotaxis is a crucial mechanism for cancer metastasis and an important mechanism for fundamental physiological processes, including angiogenesis and wound healing [16]. In this study, we will define the role of *M. oleifera* leaves and roots in terms of their mechanism of action with a thorough analysis of their content compositions. Using *D. discoideum* as a model to study the effect of ML and MR extracts for the first time has broadened our knowledge of the potential roles of these extracts. The data presented herein show that the ML extract has a significant effect on *D. discoideum* cell growth during the vegetative phase, with evidence that the chemotaxis process was delayed. These findings can be linked to the high concentration of apigenin and flavonoids in the ML extract compared to the MR extract.

## 2. Materials and Methods

### 2.1. Dictyostelium Discoideum Cell Lines and Cell Culture

*D. discoideum* axenically growing strain AX2 (wild type) was obtained from Dictybase, an online database and stock center. HL-5 medium including glucose from FORMEDIUM (Norfolk, UK) was used to induce cell growth at 22 °C. Dihydrostreptomysin from Sigma was added to the medium at a final concentration of 0.05 g/mL.

### 2.2. Source of Moringa Oleifera Leaves and Roots

Leaves and roots from four-year-old *Moringa oleifera* trees were collected at the King Faisal University Agriculture and Veterinary Research and Training Centre in Saudi Arabia.

### 2.3. Determination of Total Phenolics, Tocopherols, Flavonoids, Rutin, and Gallic Acid Through the Use of High-Performance Liquid Chromatography (HPLC)

Using one gram of the homogenized, air-dried leaves and roots of moringa plants, the concentrations of total phenolics, tocopherols, flavonoids, rutin, and gallic acid were determined using a Waters 2690 Alliance HPLC system (Waters, Milford, MA, USA) equipped with a Waters 996 photodiode array detector (Waters, Milford, MA, USA), based on the method outlined in previous studies [17,18,19,20]. The compositions found in previous studies are shown in Table 1.

### 2.4. GC/MS Analysis of Moringa oleifera Leaves and Roots

Dry air samples of Moringa oleifera leaves and roots were subjected to GC/MS analysis at King Faisal University’s Department of Chemistry and College of Science. In their study, Makkar and Becker (1996) reported on the extraction of samples using ethanol [21]. The ethanol extracts were evaluated using gas chromatography/mass spectrometry equipment (GC/MS-QP 2010 Plus) and an autosampler AOC-20i (Shimadzu, Kyoto, Japan). The materials were separated using a Restek RTX^®^-5SilMS capillary column (30 m × 0.25 mm × 0.1 µm) from Bellefonte, PA (USA). The stationary phase consisted of 5% diphenyl and 95% dimethyl-polysiloxane, with high-purity helium gas (99.9999%) serving as a carrier gas. The helium gas flow rate, sample volume, and temperature program settings were determined based on the method of El-Sherif et al., 2020 [22]. The composition was calculated based on the method of Lee, 2018, with minimal modification [23].

### 2.5. Preparation of Moringa oleifera Leaf and Root Extracts

Based on the method of Makkar and Becker (1996), the ethanolic leaf extract of *M. oleifera* was produced by blending 20 g of dried *M. oleifera* leaves and/or roots with 675 milliliters of 80% ethanol [21]. The ethanol was evaporated, and the dried extract was then resuspended in 50% DMSO solution to a final concentration of 50 mg/mL. The chemical composition of the moringa leaf and root employed in the ethanolic extract preparation process are shown in Table 1 and Table 2.

### 2.6. D. discoideum Growth Assay

The assay was applied based on a previously described method [24]. Cells were grown to reach a density of (1.5–2.5 × 10^6^ cells/mL). HL-5 medium was used to dilute the cells to 2 × 10^4^ cells/mL. Aliquots of 500 µL were then transferred to 24-well plates. The original concentration of the ML and MR extracts was 50 mg/mL in 50% DMSO as the solvent. The extracts were then diluted with HL-5 medium to reach the final concentrations of 100, 300, 900 and 1200 µg/mL. For the first control sample, the cells were incubated with DMSO at the same concentrations as that used in the plant extracts (0.1%, 0.3%, 0.9%, and 1.2%). For the second control sample, wild-type (AX2) cells were grown at the same density, 2 × 10^4^ cells/mL. Samples were incubated with the above treatments for three days at room temperature, between 21 and 22 °C. Cell density per ml was calculated using a hemocytometer over 3 days, with the experiments repeated three times.

### 2.7. Viability Assay

The viability of the treated cells was tested by mixing the cells from the samples with 0.4% trypan blue at a final concentration of 0.2%. The cells were then manually counted with a hemocytometer. Viable cells were colorless, and dead cells were blue. To calculate the percentage of viability, the live cell count was divided by the total cell count.

### 2.8. D. discoideum Development Experiment

To investigate the development phenotype, the AX2 cells were centrifuged at 700× *g* and washed with development buffer (DB) (5 mM NaH_2_PO_4_, 5mM Na_2_HPO_4_, 2 mM MgSO_4_, and 0.2 mM CaCl_2_). Cells at 2 × 10^7^/10 mL were plated onto a DB agar (1.5%) plate (90 mm dia.) containing either the ML or MR extract at a final concentration of 900 µg/mL or 1200 µg/mL. For the control samples, cells at 2 × 10^7^/10 mL were plated onto a DB agar plate (1.5%) −/+ DMSO (0.9% or 1.2%). Time-lapse imaging of the samples was carried out for 24 h using an Ikon light microscope with a SwifitCam camera (SC1803R-CK) and images were acquired every 2 min with 4× magnification at room temperature, between 20 and 22 °C. For each experiment, three samples were prepared simultaneously; the first experiment involved an ML- or MR-treated sample, which was subjected to time-lapse imaging; the second sample was the sample treated with DMSO (at the same concentration as the other treatment); and the third sample was AX2 (wt). The control samples were kept in the same area where time-lapse imaging was conducted to ensure that all samples were subjected to the same conditions. Experiments were repeated at least twice.

### 2.9. Statistical Analysis

A Student’s *t*-test was employed to analyze statistical significance using Microsoft Excel.

## 3. Results

### 3.1. HPLC Analysis for Moringa oleifera Roots and Leaves Extracts

The secondary metabolites shown in Table 1 include polyphenols, flavonoids, tocopherols, rutin, and chlorogenic acid. The data show that the rutin compound is absent in the Moringa oleifera roots; in comparison, the Moringa leaves contain greater concentrations of total apigenin and flavonoids.

### 3.2. GC/MS Analysis of the Leave and Roots of Moringa oleifera

The phytochemical composition of ethanol extracts from *Moringa oleifera* leaves and roots is shown in Table 2 and Supplementary Tables S1 and S2. It is recognized that different compounds in *M. oleifera* possess specific functions [15]. The data presented herein show that some of the bioactive compounds were found only on the *M. oleifera* leaves, namely ethyl octanoate (ester compound), methoxy-5-aminophenol (phenol derivative), decanoic acid (fatty acid), and fenchol (terpene). Moreover, ethyl palmitate, ethyl linolenate, palmitic acid, and 2-(octadecyloxy) ethyl ester were found at elevated levels in the leaf extract. However, the compounds methylbenzyl alcohol (alcohol), phenyl-1-butene (hydrocarbon), and para-chloro-meta-xylenol (PCMX) (a chlorinated phenolic antiseptic agent) were found only in the roots. In addition, elevated levels of benzylamine and elaidic acid were found in this extract.

Some of the bioactive compounds were found in both extracts, including benzylamine (amine), lauric acid (fatty acid), palmitic acid (fatty acid), and ethyl linoleate (linoleic acid esters).

### 3.3. Effect of ML and MR Extracts at Different Concentrations on D. discoideum Cell Growth

The biological effect of ML and MR extracts on cell growth was assessed by using *D. discoideum* wild-type axenic strain AX2 cells. As described in Section 2.6, cells were grown in HL-5 media on a 24-well plate at a density of 2 × 10^4^/mL per well. The cells were then treated with different concentrations of ML and MR extracts (100, 300, 900, and 1200 µg/mL). Next, the cells were counted using a hemocytometer over a period of three days. For the control samples, the cells were treated with DMSO at final concentrations of 0.1%, 0.3%, 0.9%, and 1.2%, which were used as solvents for the *M. olefira* extracts.

**Figure 1:**
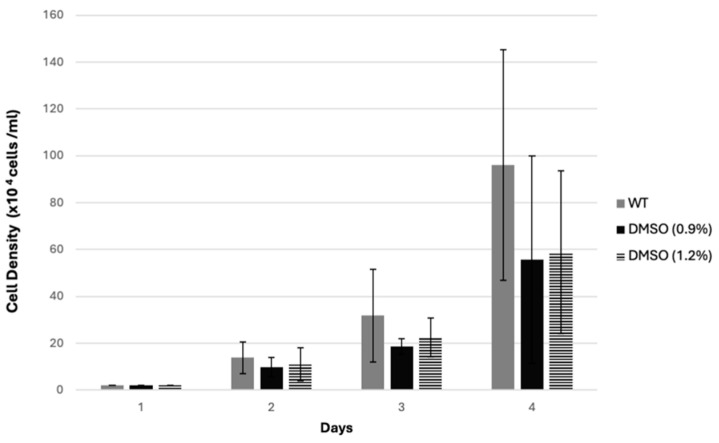
The effect of DMSO on *D. discoideum* cell growth. AX2 cells were grown at density of 2 × 10^4^ cells/mL in a 24-well plate, treated with different concentrations of DMSO, and then counted for three days, as described in Section 2.6. The graph shows the effect of DMSO at the highest concentrations, 0.9% and 1.2%, on AX2 cell growth. The values represent the mean of three independent experiments ± SD.

We first investigated whether the solvent had a significant effect on cell growth compared to the WT sample. As shown in Figure 1, the DMSO-treated samples at the highest concentrations (0.9% and 1.2%) exhibited no significant effect on cell growth compared to the untreated samples (Figure 1).

**Figure 2:**
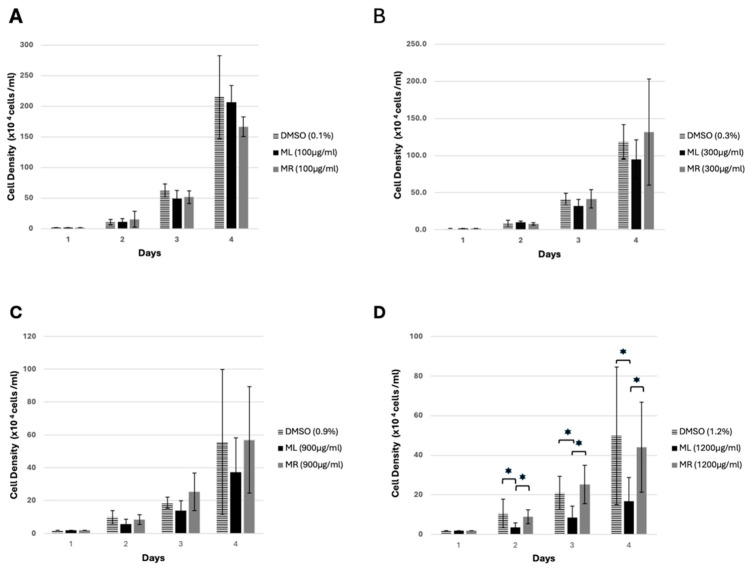
Cell growth of *D. discoideum*. AX2 cells were grown on a 24-well plate as described in Section 2.6. The graph shows the effect of ML and MR extracts at final concentrations of 100 µg/mL (**A**), 300 µg/mL (**B**), 900 µg/mL (**C**), and 1200 µg/mL (**D**) on AX2 cell growth over a 72 h incubation period. The DMSO-treated samples were used as controls for each experiment. The values represent the mean of three independent experiments ±SD (* *p* < 0.05).

**Figure 3:**
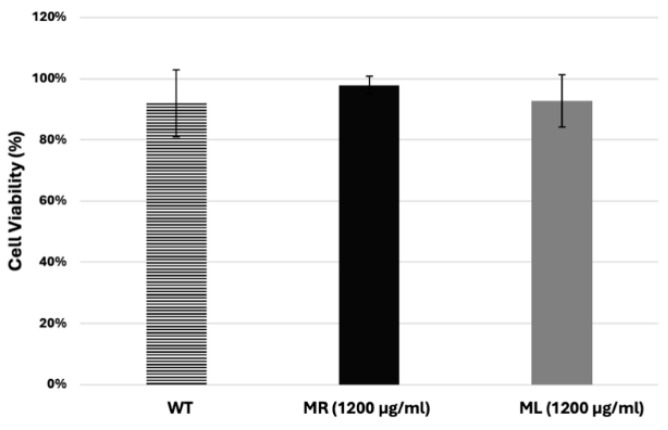
Viability percentage of *D. discoideum* cells. A viability assay was performed, as described in Section 2.7. The graph shows the percentage of viability for cells treated with the ML and MR extracts at the highest concentration, 1200 µg/mL, over a period of 72 h compared to the AX2 (WT) cells.

The data also showed that the ML and MR extracts exhibited no growth inhibition activity at concentrations of 100 µg/mL, 300 µg/mL, and 900 µg/mL (Figure 2A–C). However, treating the cells with the ML extract at a final concentration of 1200 µg/mL resulted in a significant inhibition of cell growth after 24 h incubation compared to the MR extract-treated sample and control (Figure 2D). The inhibition of cell growth was not the result of cell death, as the viability assay showed no differences between the treated samples compared to the control (Figure 3).

### 3.4. Effect of ML and MR Extracts at Concentrations of 900 µg/mL and 1200 µg/mL on D. Discoideum’s Development Life Cycle

**Figure 4:**
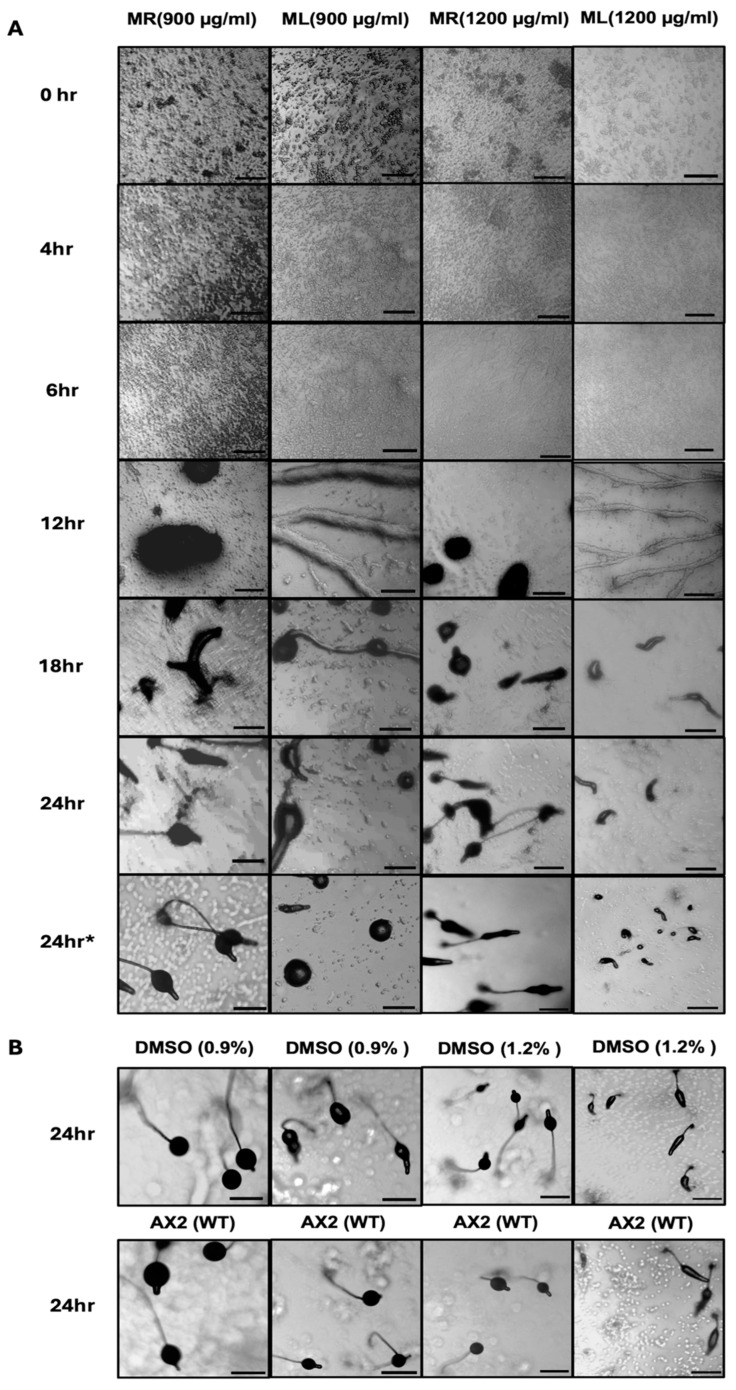
Time-lapse imaging for the development cycle of *D. discoideum* cells on a DB agar (1.5% agar) plate at a density of 2 × 10^7^ cells per plate (90 mm dia.). Images were captured every 2 min with 4× magnification. (**A**) Phenotype for the development cycle of AX2 cells on the DB agar plate containing MR or ML extracts at a concentration of either 900 µg/mL or 1200 µg/mL for 24 h. (**B**) Phenotype for 24 h developed control samples that were performed in parallel with the experiments in part (**A**) and under the same conditions. Images for control samples show the completion of the development life cycle of AX2 cells on the DB agar plate +/− the solvent DMSO at the final concentrations of 0.9% and 1.2%. * Different field of view for the same sample which was used for the time-lapse imaging. Scale bar is 200 µm.

*D. discoideum* cells show a unique development phase upon the commencement of starvation. This phase starts with the cell aggregation stage and ends with multicellular fruiting body formation after 24 h. The aggregation process is dependent on cell streaming and chemotaxis toward cAMP. To examine the effect of *M. oleifera* extracts on cell chemotaxis and streaming, the cells were washed with DB and starved onto a DB agar plate including either the ML or MR extract at a final concentration of either 900 µg/mL or 1200 µg/mL. Two control samples were used in this experiment. Time-lapse imaging was applied for 24 h for each treated sample independently (Figure 4A). The control samples for each experiment including DMSO-treated sample and untreated sample were prepared, concurrently with the treated samples. In addition, during time-lapse imaging for treated samples, the control samples were subjected to the same conditions as the sample undergoing imaging. After the treated cells completed 24hr starvation, time-lapse imaging was stopped and images for control samples were captured to check the completion of cells development life cycle (Figure 4B). The data showed that the ML extract-treated samples at concentrations of 900 µg/mL and 1200 µg/mL exhibited a noticeable delay in development life cycle completion. The delay started during the aggregation stage; however, cell streaming does not appear to be affected in ML extract-treated cells (Figure 4A). The delay was not observed with MR extract treatment at the concentrations of 900 µg/mL and 1200 µg/mL (Figure 4A). In addition, no differences were observed between the development life cycle of AX2 cells and the control samples (1.5% agar + 0.9% DMSO) or (1.5% agar + 1.2% DMSO) (Figure 4B). These data indicated that the solvent (DMSO) had no significant effect on the development life cycle of the *D. discoideum* cells.

## 4. Discussion

*Moringa. oleifera* represents one of the plants commonly used in research, considered a medicinal compound that is rich in beneficial components [25]. Different parts of the plant and their uses have been subjected to evaluation in the biomedical research field. In a study, Moringa seed extract induced a reduction in SH-SY5Y human neuroblastoma cell growth and exhibited inhibition of NF-κB signaling [26]. Moreover, apoptosis was induced in human lung cancer cells treated with Moringa leaf extract [27]. Different parts of this plant are considered beneficial compounds in the treatment of numerous diseases including neurodegenerative, cancer and ulcers [3,28]. There is value in identifying new natural compounds in different parts of this plant and specifying the importance of this plant in the biomedical field.

In this study, we used leaf and root extracts from the *M. oleifera* plant, which grows in the eastern part of Saudi Arabia, to determine their biological effect on *D. discoideum* cells. This cellular model has been widely used to study the potential functions of different natural components, avenues in drug development, and the underlying molecular mechanisms [14,29,30]. The distinct development life cycle of this model and the ability of cells to undergo chemotaxis make it a widely used tool in research on cancer and neurological disorders [31,32]. Given that different compositions have been detected for different *M. oleifera* plants that grow in different regions, we aimed to investigate the active components that plants can possess [33]. HPLC analysis results showed elevated flavonoid and apigenin levels in the ML extract compared to the MR extract. Flavonoids including Apigenin are the largest group of polyphenols. It is known that Flavonoids act as antioxidants and showed anti-proliferation activity, while Apigenin plays a role in regulating apoptotic and anti-inflammatory pathways and cause cell cycle arrest [34]. The high level of flavonoids is consistent with previous research regarding *M. oleifera* leaves. The elevation in apigenin levels could be an indication of the importance of the plant used in this study. Based on our CG-MS analysis results, Ethyl palmitate and Ethyl linolenate represent the two components that were found at elevated levels in the ML extract. These components are both fatty acid ethyl esters (FAEEs). As indicated in previous studies, ethyl palmitate impacts the cell cycle and exhibits anti-inflammatory activity [35,36]. *D. discoideum* cells treated with the ML extract at a concentration of 1200 µg/mL exhibited significantly decreased cell growth. However, this effect was not detected in the MR extract-treated sample at the same concentration. These data are consistent with the results of previous studies employing such treatment but with different cell models [5,6]. The data from the viability assay confirmed that cell growth inhibition in the ML extract-treated samples is not related to cell apoptosis. The elevation of flavonoids levels could be one factor responsible for this effect based on a wide range of evidence on the health benefits of this large family of polyphenolics, including anticancer agents [37]. In a study conducted by Waheed et al. 2013, the results showed that naringenin, a dietary flavonoid, exhibited antiproliferative activity in *D. discoideum* cells in TRPP2 (polycystin-2)-dependent manner [38]. Furthermore, the high level of apigenin in the ML extract compared to the MR extract could also be a strong contributer for cell growth reduction. Based on the results of a previous study, apigenin may exhibit anticancer activity [39]. As chemotaxis is a major cause of cancer metastasis, we investigated the effect of ML and MR extracts on cell-directed migration by applying time-lapse imaging during the development life cycle of *D. discoideum* cells. The addition of the ML extract showed a noticeable delay in the aggregation stage, which led to a delay in development life cycle completion. This effect was detected with both concentrations, 900 µg/mL and 1200 µg/mL. In contrast with the ML extract-treated samples, the MR extract-treated cells were able to proceed through the development life cycle in a similar manner to the controls. In *D. discoideum* cells, different molecular mechanisms can be involved in the aggregation stage. It has been shown that Ca^2+^ concentration plays a role in cAMP production. Moreover, cell cytoskeleton including actin plays a major role in cell proliferation and cell movement by different actin-binding proteins [40,41,42]. It is possible that the ML extract affects cell growth and cell aggregation indirectly through one of the indicated molecular mechanisms. Cell chemotaxis has also been linked to the development of neurodegenerative diseases [43]. Furthermore, the human CLN3 protein is associated with Neuronal Ceroid Lipofuscinosis (NCL), which is one of the most common types of neurodegenerative disorders in children. It has been established that the genome of *D. discoideum* encodes an ortholog of human CLN3 [44,45]. Loss of function of the Cln3 protein in *D. discoideum* cells caused a delay in the aggregation stage in a similar manner as that observed in the ML extract [45]. For this, Cln3 protein can be a possible target for ML extract in *D. discoideum* cells, where a neuroprotective role has been noted for *M. oleifera*, and it has been highlighted as a potential form of treatment for neurodegenerative diseases [46]. The extract from the leaves showed promising results for the treatment of Alzheimer’s disease, and the role of apigenin has been linked to neuroinflammation [47,48]. Although the traditional uses of this plant require further exploration, the data presented herein can provide a future direction for studying the impact of the biological activity of Moringa leaves on neurodegenerative disorders. Further research is needed to clarify the ability of cells to respond to the cAMP and whether Ca^2+^ concentration and the dynamic reorganization of actin have changed after ML extract treatment. In addition, the roles of apigenin and the underlying molecular mechanism need to be undertaken.

## 5. Conclusions

The results of this study using slime mold *D. discoideum* as cellular model indicated that the ML extract significantly reduces cell growth and causes a delay in the cell aggregation stage of the development cycle. However, this effect was not detected using MR extract. The elevation level of flavonoids and apigenin in ML extracts, which, generated by HPLC analysis, may contribute to this action.

## Figures and Tables

**Table 1:** Chemical composition of *Moringa oleifera* leaves and roots using the HPLC method.

Chemical	Amount		Unit
	Leaf	Root	
Total apigenin	10.5	2.65	µmol/g
Total polyphenols	28.1	29.3	mg/g
Total flavonoids	81	1.53	mg/g
Total tocopherols	5	5.36	μg/g
Total rutin	0.278	Absent	mg/g
Total gallic acid	0.128	0.09594	mg/g

**Table 2:** Phytochemical composition of ethanol extracts from *Moringa oleifera* root and leaves based on GC MS analysis.

Essential Oil Compounds	Area %	
	Root	Leaves
Benzylamine	29.28	0.02
2,3-Dihydro-2,5-dihydroxy-6-methyl-4*H*-pyran-4-one	0.17	0.02
2-Methyl-1-butene	1.55	0.09
Lauric acid	0.12	0.09
Dibutyl phthalate	0.1	0.05
Palmitic acid	7.8	1.23
Ethyl stearate	0.67	7.15
Ethyl Linoleate	1.03	4.73

## Data Availability

The original contributions presented in this study are included in the article/Supplementary Material. Further inquiries can be directed to the corresponding author.

## Supplementary Materials

The following supporting information can be downloaded at: https://www.mdpi.com/article/10.3390/biology14030284/s1, Table S1. Phytochemical composition of ethanol extracts from *Moringa oleifera* leaves based on GC MS analysis. Table S2. Phytochemical composition of ethanol extracts from *Moringa oleifera* root based on GC MS analysis.
