# Supplementary material for: Characterizing the Role of Moringa oleifera Lam (MO) Leaves and Root Extracts on Dictyostelium discoideum Cell Behavior
Source: Biology (Basel). 2025 Mar 11;14(3):284. doi: 10.3390/biology14030284 (PMC11940813; doi:10.3390/biology14030284)
Supplement: Supplementary file 1 [file biology-14-00284-s001.zip › biology-3444015-supplementary.pdf]

biology

MDPI

Article

Characterizing the Role of *Moringa Oleifera* Lam (MO) Leaves and Root Extracts on *Dictyostelium discoideum* Cells Behavior

Sarah Abdulaziz Alamer * and Fadia El-Sherif

Department of Biological Sciences, College of Science, King Faisal University, AlAhsa 31982, Saudi Arabia.
* Correspondence: salamer@kfu.edu.sa

Table S1. Phytochemical composition of ethanol extracts from *Moringa oleifera* leaves based on GC MS analysis.

Peak	RT, min	Area, %	Essential oil compounds	Molecular weight (g·mol−1)	Molecular formula
1	9.27	0.02	Benzylamine	107.15	C7H9N
2	9.98	0.01	Nonanal	142.24	C9H18O
3	11.028	0.02	2,3-Dihydro-2,5-dihydoxy-6-methyl-4-H-pyran-4-one	144.12	C6H8O4
4	12.306	0.09	2-Methyl-1-butene	70.13	C5H10
5	12.557	0.03	Ethyl octanoate	172.26	C10H20O2
6	13.427	0.06	2-Methoxy-5-aminophenol	139.15	C7H9NO2
7	14.109	0.04	2-Decenal	154.25	C10H18O
8	14.351	0.02	Decanoic acid	172.26	C10H20O2
9	15.364	0.03	Fenchol	154.25	C10H18O
10	16.852	0.1	2-Furanmethanol, 5-ethenyltetrahydro-alpha,alpha,5-trimethyl-, (2R,5R)-rel-	170.25	C10H18O2
12	19.512	0.17	Dihydroactinidiolide	180.24	C11H16O2
13	20.181	0.09	lauric acid	200.32	C12H24O2
14	20.253	0.05	2-Mercaptopyridin-3-ol	127.17	C5H5NOS
15	20.613	0.09	Diethyl phthalate	222.24	C12H14O4
16	21.33	0.23	Methyl beta-D-glucoside	194.18	C7H14O6
17	21.493	0.07	1-Naphthalenemethanol, 1,4,4a,5,6,7,8,8a-octahydro-2,5,5,8a-tetramethyl-	222.37	C15H26O
18	22.031	0.33	Azelaic acid	188.22	C9H16O4
19	22.219	0.07	(Z)-9-Tetradecenal	210.36	C14H26O
22	26.272	0.05	Ethyl tetradecanoate	256.42	C16H32O2
23	26.576	0.06	Pentadecane	212.41	C15H32
24	28.307	2.3	Fitone	268.5	C18H36O
25	31.637	0.07	Hexadecane	226.44	C16H34
26	32.254	0.04	Lauryl glycidyl ether	242.4	C15H30O2
27	32.549	0.26	(Z)-7-Hexadecenal	238.41	C16H30O
28	34.654	0.05	Dibutyl phthalate	278.34	C16H22O4
29	36.635	1.23	Palmitic Acid	256.42	C16H32O2
30	38.078	0.63	Ethyl 9-hexadecenoate	282.5	C18H34O2
31	39.005	27.32	Ethyl palmitate	284.5	C18H36O2
32	44.258	0.45	Ethyl heptadecanoate	298.5	C19H38O2
33	44.557	3.67	Phytol	296.5	C20H40O
34	45.158	1.01	dodec-7-enyl acetate	226.35	C14H26O2
35	45.307	4.58	9,12,15-Octadecatrienoic acid, methyl ester, (9Z,12Z,15Z)	292.5	C19H32O2
36	45.531	0.47	Hexadecanal	240.42	C16H32O

37	45.751	4.73	Ethyl Linoleate	308.5	C20H36O2
38	45.874	14.68	Ethyl linolenate	306.5	C20H34O2
39	46.046	0.61	Ethyl oleate	310.5	C20H38O2
40	46.158	0.77	(Z)-7-Hexadecenal	238.41	C16H30O
41	46.509	7.15	Ethyl stearate	312.5	C20H40O2
42	47.632	3.9	(Z)-9-Tricosene	322.6	C23H46
43	49.565	0.32	6-Hexyltetrahydro-2H-pyran-2-one	184.27	C11H20O2
44	49.763	1.13	alpha-Caryophyllene oxide	220.35	C15H24O
45	49.883	0.45	Steviol	318.4	C20H30O3
46	50.169	0.8	2(3H)-Benzofuranone, hexahydro-4,4,7a-trimethyl	182.26	C11H18O2
47	50.268	0.68	alpha-Linoleic acid	280.4	C18H32O2
49	50.942	0.64	Spiro[4.5]decane	138.25	C10H18
51	52.018	0.14	2-Isopropenyl-4a,8-dimethyl-1,2,3,4,4a,5,6,8a-octahydronaphthalene	204.35	C15H24
52	52.585	0.18	1H-Cycloprop[e]azulene, 1a,2,3,5,6,7,7a,7b-octahydro-1,1,4,7-tetra-methyl-, [1aR-(1aalpha,7al-pha,7abeta,7balpha)]	204.35	C15H24
54	54.431	15.86	Palmitic acid, 2-(octadecyloxy)ethyl ester	553	C36H72O3

Table S2. Phytochemical composition of ethanol extracts from *Moringa oleifera* root based on GC MS analysis.

Peak	RT, min	Area, %	Essential oil compounds	Molecular weight (g·mol−1)	Molecular formula
1	6.893	29.28	Benzylamine	107.15	C7H9N
2	10.401	1.47	4-Methylbenzyl alcohol	122.16	C8H10O
3	11.024	0.17	2,3-Dihydro-2,5-dihydoxy-6-methyl-4-H-pyran-4-one	144.12	C6H8O4
4	13.891	1.55	2-Methyl-1-butene	70.13	C5H10
6	15.26	1.46	4-Benzyloxybenzonitrile	209.24	C14H11NO
7	16.087	1.12	1-Benzyl-3-pyrrolidinone	175.23	C11H13NO
8	16.304	2.26	4-PHENYL-1-BUTENE	132.2	C10H12
9	16.784	0.42	para-chloro-meta-xylenol (PCMX)	156.61	C8H9ClO
10	16.938	0.45	2-Phenylacetamide	135.16	C8H9NO
11	17.044	0.44	N-benzylformamide	135.16	C8H9NO
12	17.42	0.09	1,3-Diphenylacetone	210.27	C15H14O
13	17.651	0.39	1,4-Diacetylbenzene	162.18	C10H10O2
14	17.767	1.86	N-Acetylbenzylamine	149.19	C9H11NO
16	19.162	0.1	2,4-Di-tert-butylphenol	206.32	C14H22O
17	19.219	0.13	Glycine, N-(phenylmethyl)-, ethyl ester	193.24	C11H15NO2
18	20.172	0.12	Lauric acid	200	C12H24O2
19	20.274	0.45	Benzyl bromide	171.03	C7H7Br
20	20.495	0.2	2-Phenylethyl bromide	185.06	C8H9Br
21	20.611	0.12	Diisobutyl phthalate	278.34	C16H22O4
22	20.771	0.06	Ethyl dodecanoate	228.37	C14H28O2
23	21.313	0.2	Methyl alpha-D-glucopyranoside	194.18	C7H14O6
25	21.778	0.68	3-Methyl-1-phenyl-1H-pyrazol-5-amine	173.21	C10H11N3
27	24.391	4.35	N-Benzylidenebenzylamine	195.26	C14H13N
28	25.024	0.1	Myristic acid (Tetradecanoic acid)	228.37	C14H28O2
29	25.564	0.49	1-Benzyl-2-pyrrolidinone	175.23	C11H13NO
30	26.002	0.28	N-Benzylethylenediamine	150.22	C9H14N2
31	33.19	0.18	Methyl palmitate	270.5	C17H34O2
32	34.651	0.1	Dibutyl phthalate	278.34	C16H22O4
33	36.303	7.8	Palmitic acid	256.42	C16H32O2
35	38.739	0.67	Ethyl stearate	312.5	C20H40O2
36	43.174	0.07	Heptadecanoic acid	270.5	C17H34O2
39	44.297	0.49	Methyl oleate	296.5	C19H36O2

40	45.188	8.93	9,12-Octadecadienoic acid	280.4	$C_{18}H_{32}O_2$
41	45.396	17.28	Elaidic Acid	282.5	$C_{18}H_{34}O_2$
42	45.736	1.03	Ethyl Linoleate	308.5	$C_{20}H_{36}O_2$
43	45.909	5.42	Stearic acid	284.5	$C_{18}H_{36}O_2$
44	46.492	0.42	Octadecanoic acid, ethyl ester	312.5	$C_{20}H_{40}O_2$
46	48.374	0.17	cis-9-Tricosene	322.6	$C_{23}H_{46}$
48	50.193	0.1	Arachidic Acid	312.5	$C_{20}H_{40}O_2$
49	54.091	4.37	1-Hexacosanol	382.7	$C_{26}H_{54}O$
